# Free Chlorine Can Inhibit Lead Solder Corrosion via Electrochemical Reversal

**DOI:** 10.1021/acs.est.4c07375

**Published:** 2024-10-18

**Authors:** Frank A. Mazzola, Kathryn G. Lopez, Marc Edwards

**Affiliations:** The Charles Edward Via, Jr. Department of Civil and Environmental Engineering, Virginia Tech, Blacksburg, Virginia 24061, United States

**Keywords:** drinking water, lead solder, chlorine, galvanic corrosion, electrochemistry

## Abstract

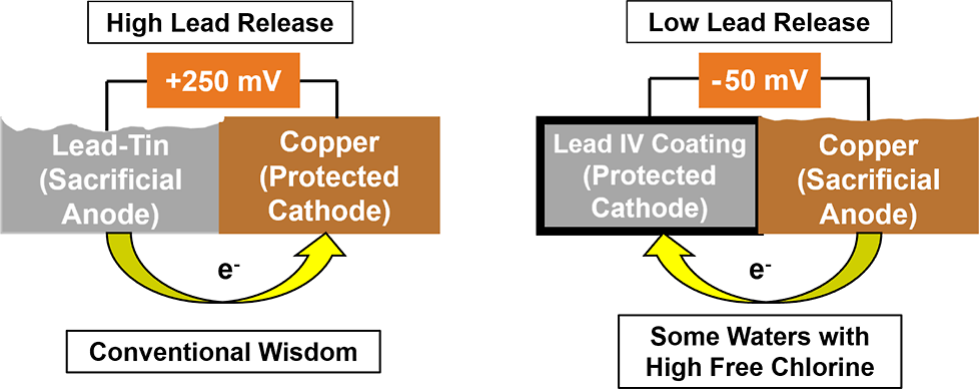

Galvanic corrosion of lead–tin solder in copper plumbing can be a major contributor to water lead contamination. Here, we report the electrochemical reversal of the copper-solder galvanic couple, in which the normally anodic solder becomes cathodic to copper via a reaction with free chlorine. This reversal occurred after a few months of exposure to continuously circulating water with relatively low pH and low alkalinity, causing dramatically decreased lead release and the formation of a Pb(IV) scale. Chloramine did not similarly inhibit solder corrosion over the 4–9 month test duration, resulting in up to 100 times more lead contamination of the water relative to free chlorine. These findings have major implications for corrosion control and public health and can help explain anomalously low levels of lead contamination in some waters with free chlorine that are normally considered corrosive to solder.

## Abstract 2

After months of continuous flow, high levels of free chlorine disinfectant drastically reduced water lead contamination by causing initially anodic lead−tin solder to become cathodic to copper pipe.

## Introduction

In a conventional lead–copper galvanic cell, the copper cathode is protected from corrosion, while the anodic lead is sacrificed. The physical connection of lead pipe to copper pipe in drinking water, or the deposition of copper on lead pipe surfaces, can therefore increase water lead contamination.^1−7^ When lead pipe undergoes prolonged exposure to free chlorine, a Pb(IV) scale can sometimes form that elevates the corrosion potential of the lead to a point it becomes cathodic to copper and electrochemically protected from galvanic corrosion (i.e., electrochemical reversal).^8^ Chloramine does not form a Pb(IV) scale on lead pipe associated with electrochemical reversal, allowing sacrificial galvanic currents and lead contamination of water to be sustained indefinitely.^8−10^ These observations can explain a few cases of serious water lead contamination observed at utilities switching from free chlorine to chloramine disinfectant.^1,8,9,11−14^

The electrochemical reversal of Pb/Sn solder alloys that were used to join copper pipes in most home plumbing until 1986 has never been previously documented. If it were to occur, it could help explain why a few utilities switching from free chlorine to chloramine without any known lead pipe have reported sudden elevations in water lead due to solder corrosion.^15−17^ Here, we attempt to unambiguously demonstrate free chlorine-induced electrochemical reversal of the lead–tin solder and copper pipe galvanic couple using laboratory synthesized waters with low alkalinity. For comparison, waters were tested with chloramine, phosphate addition, and a range of sulfate and pH levels. Lead release was tracked by using simulated lead solder-copper pipe joints. Electrochemical trends were monitored in real time via Pb/Sn solder wires externally connected electrically to a copper pipe, and the mineralogical composition of the resulting pipe scales was examined.

## Methods

### Experimental Apparatus

**Figure 1:**
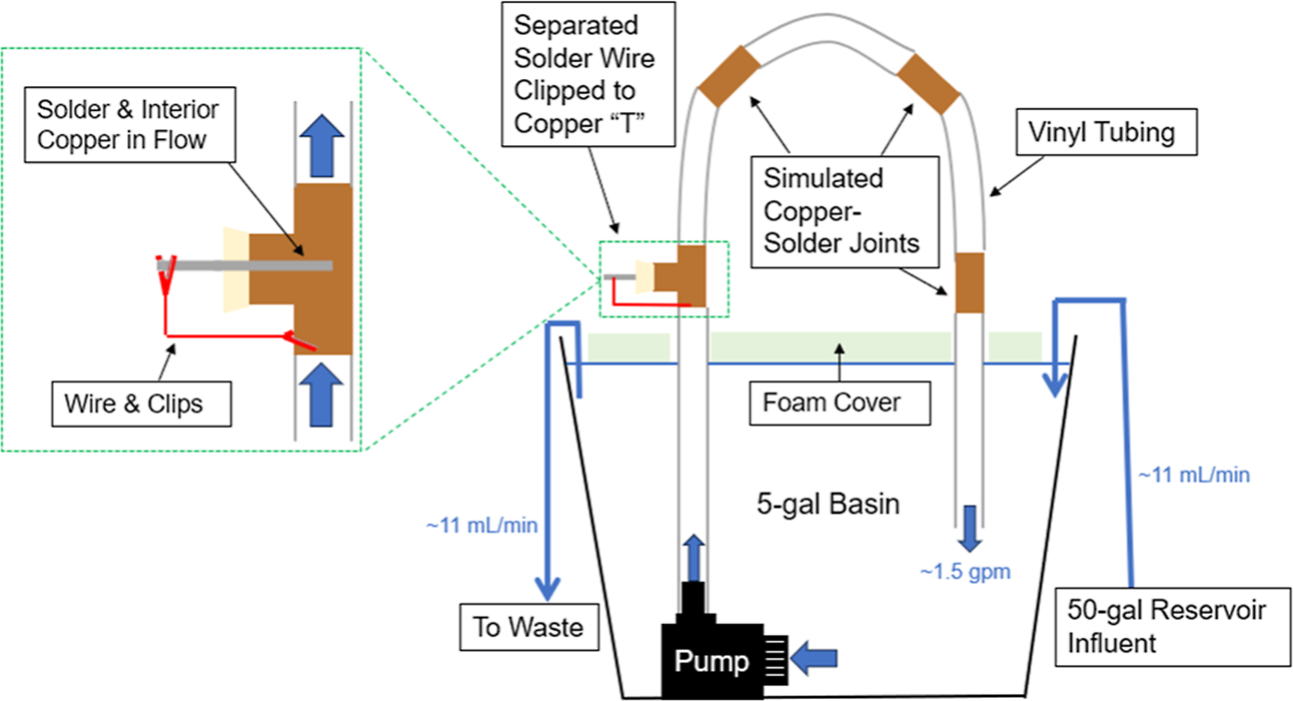
Pipe-loop apparatus.

Continuously flowing pipe-loops were constructed to monitor aspects of electrochemical reversal and water lead contamination (Figure 1). Each pipe-loop was fed from a 5 gal basin with a continuously flowing pump at ∼1.5 gpm. The pipe loops each included three simulated copper-solder joints and one physically separated but galvanically connected copper-solder couple. The simulated joints were made by melting 1 ± 0.05 g of 50:50 Pb/Sn solder wire onto a 2-in-long segment of 1/2-in. diameter copper pipe, similar to methods used elsewhere.^18^ On the other hand, the separated copper-solder couples were constructed by inserting a 4 ± 0.15 g segment of 50:50 lead–tin solder wire through a rubber stopper, which was placed into a 1/2-in. diameter copper “*T*” joint (Figure 1). The solder wire and copper *T* were then connected by external jumper wires to allow for regular measurement of galvanic current, voltage, and electrochemical corrosion potential (*E*_corr_) of each metal during flow as described elsewhere.^8^

The basin was mostly closed to the atmosphere with a floating foam cover. To maintain a stable free chlorine residual of ∼4 mg/L as Cl_2_, as well as stable pH and water composition, peristaltic pumps dosed the appropriate target water with elevated chlorine at a rate of ∼11 mL/min from a 50-gallon reservoir, and excess water from the system overflowed to waste (Figure 1). This high chlorine dose and the continuous flow conditions were selected based on the belief that they would accelerate the heretofore never previously reported electrochemical reversal of the Pb/Sn solder-copper galvanic couple. Due to the slower decay rate of chloramine, it was sufficient to replace the water in the 5-gallon reservoir ∼3 times per week and maintain a total chlorine residual of ∼3–4 mg/L.

### Experimental Phases and Measurements

Testing proceeded in two phases, each with a new batch of simulated soldered joints. Prior to beginning the phase 1 study, copper-solder joints were conditioned for 1 week in “water A” (Supporting Information, Table S1) with low alkalinity and high chloride-to-sulfate mass ratio (CSMR) using a static dump-and-fill method with daily water changes. A pH of 8.3 was used for phase 1 conditioning and a pH of 7.3 was used for phase 2 conditioning. Collected effluent was analyzed for lead release using a Thermo Electron iCAP RQ inductively coupled plasma–mass spectrometer. Simulated joints with similar lead release (relative standard deviation < 20%) were selected in groups of three replicates that had statistically similar means and standard deviations (Supporting Information Table S2). For phase 1 testing, the mean lead release for each set of triplicates was 1240-1290 ppb. Likewise, the batches of simulated joints used for phase 2 testing had mean lead release of 1090-1110 ppb. The electrochemistry measurements (current, voltage, and corrosion potential) were taken at the separated copper-solder couple approximately once per week. An Ag–AgCl reference electrode was used for the electrochemical corrosion potential (*E*_corr_) measurements.

After >8 months of exposure during phase 1, and 4 months of exposure during phase 2, the copper-solder joints were removed from the test rigs to be analyzed for lead release during stagnation. Each simulated joint was placed into a 125 mL bottle and the water was changed daily. Following a two-week stabilization period, the effluent water was collected on six consecutive days. After phase 2, lead release from the lengths of solder wires that were exposed to flow as part of the separated galvanic couple was determined by placing the wire segment into 20 mL of stagnant water without any connection to copper. This test was conducted to determine lead release from solder without any galvanic effect at the same solder surface area to water volume ratio tested for the simulated joints. The water was collected every 24 h for six consecutive days following a two-week stabilization period. Following phase 1, *E*_corr_ measurements of the solder and copper surfaces on the simulated joint were made during stagnation using a micro-Ag-AgCl electrode, similar to microelectrode methods described elsewhere.^19,20^ Galvanic voltage during stagnation was determined by the difference between copper and solder *E*_corr_ measurements, using a convention that a positive voltage indicates that the solder is anodic to copper. Thereafter, the surfaces of select joints were analyzed using scanning electron microscopy (SEM; FEI Quanta 600 FEG operated at 30 kV). The scale of select samples were then scraped and ground, and corrosion solids were characterized by X-ray diffraction (Wide-Angle Bruker D8 XRD operated at 40 kV and 40 mA). Scans were conducted over the range of 20–62° 2θ, with 0.04-degree step sizes that were each 1 s long. Reference XRD patterns were obtained from the International Centre for Diffraction Data.

### Water Conditions

Various water conditions were tested using two synthesized water types A and B (Supporting Information, Table S1) with low alkalinity, low hardness, and high CSMR. The water was prepared by adding sodium metasilicate nonahydrate, magnesium sulfate, and calcium chloride dihydrate to deionized water. Sodium hypochlorite solution was used to add chlorine, and ammonium hydroxide was dosed as necessary to form chloramine at a 4:1 mass ratio of chlorine to ammonia. Waters A and B were modified to explore a range of pH levels, CSMRs, and orthophosphate corrosion control (Table 1, Supporting Information, Table S3). Water chemistry was monitored ∼3 times per week, with pH and chlorine levels adjusted as needed. Some conditions were tested with extra sulfate to evaluate lower CSMRs. The alkalinity targets for waters A and B were 12 and 17 mg/L as CaCO_3_, respectively. The chemistries of these low alkalinity waters were selected to be somewhat similar to that used in prior studies in Portland, Oregon, where lead release from soldered copper joints decreased dramatically after a few months exposure to chlorinated (but not chloraminated) water.^21^

**Table 1:** Primary Water Conditions and Target Water Quality Parameters

test water name	water type	pH	chlorine (mg/L as Cl2)	ammonia (mg/L-N)	approx. CSMR
phase 1					
water A, chlorine, pH 8.3	A	8.3	4.0	0	10
water A, chlorine, pH 9.3	A	9.3	4.0	0	10
water A, chloramine, pH 8.3	A	8.3	4.0	1.0	10
water A, control, pH 8.3	A	8.3	0	0	10
phase 2					
water B, chlorine, pH 7.9	B	7.9	4.0	0	2
water B, chloramine, pH 7.9	B	7.9	4.0	1.0	2
water A, chlorine, pH 7.3	A	7.3	4.0	0	10
water A, chlorine, pH 7.3, CSMR 0.5	A	7.3	4.0	0	0.5
water A, chloramine, pH 7.2	A	7.2	4.0	1.0	10

### Statistics

For analysis of metal release from simulated joints during phase 2, pooled *t* tests were used based on two consecutive 3-day composite collections for each replicate (*n* = 3) using an alpha value of 0.05. For the separated solder wires, each daily collection was analyzed separately, and an alpha value of 0.05 was also used.

## Results and Discussion

### Electrochemical Corrosion Potential and Voltage During Flow

**Figure 2:**
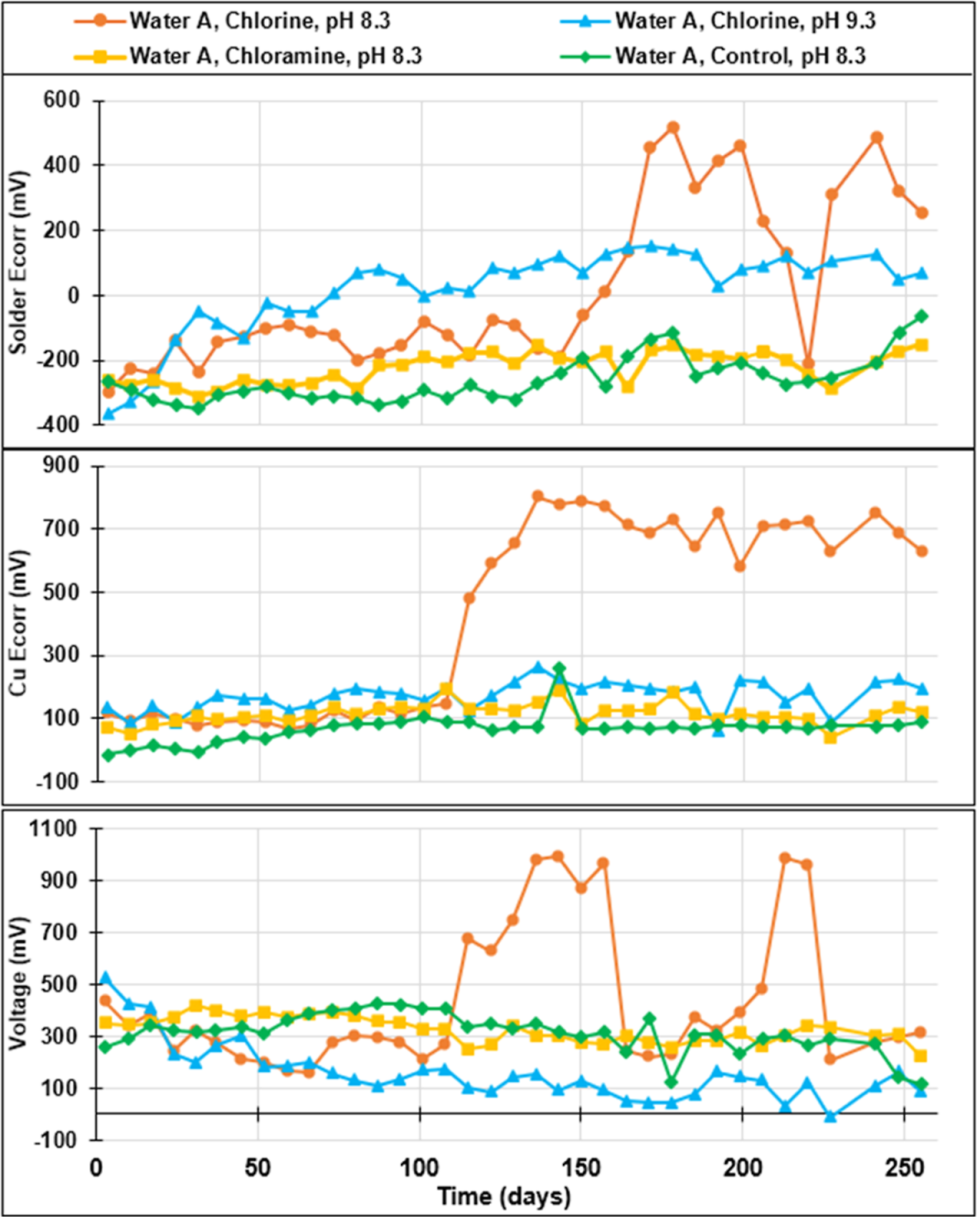
Phase 1 electrochemical corrosion potential (*E*_corr_ vs AgCl) and galvanic voltage measured during flow at separated copper-solder couple.

Phase 1 tests were focused on higher pH levels that were previously demonstrated to cause rapid electrochemical reversal for pure lead.^8^ At the start of the testing, lead solder *E*_corr_ values ranged from −400 to −200 mV versus an AgCl reference electrode for all water conditions (Figure 2). The *E*_corr_ of the solder exposed to free chlorine increased steadily over a period of months to a maximum of +518 mV for the chlorine-treated condition at pH 8.3, similar to trends reported by Arnold and Edwards for pure lead.^8^ Likewise, the lead–tin solder *E*_corr_ increased to +150 mV for the chlorine-treated condition at pH 9.3. As predicted, positive lead *E*_corr_ values were never recorded for any conditions with chloramine or without any chlorine.

Full electrochemical reversal, as defined by the *E*_corr_ of solder exceeding that of copper to produce a negative voltage, was never observed at pH 8.3 during flow because of a massive rise in copper *E*_corr_ to +802 mV. Waters with high free chlorine, low alkalinity, and high pH are known to demonstrate a propensity for copper pitting, which can cause such dramatic rises in copper *E*_corr_.^22−26^ Electrochemical reversal was also never recorded for the condition with orthophosphate (Supporting Information, Figure S1), similar to reports of Arnold and Edwards for pure lead,^8^ and work of Lytle et al. and others indicating that orthophosphate may inhibit Pb(IV) formation.^27^ Electrochemical reversal was documented, but only on day 227 for the condition at pH 9.3 with high CSMR (Figure 2) and on two other days for the chlorinated condition at pH 9.3 with extra sulfate to reach a CSMR of 0.5 (Supporting Information, Figure S1).

**Figure 3:**
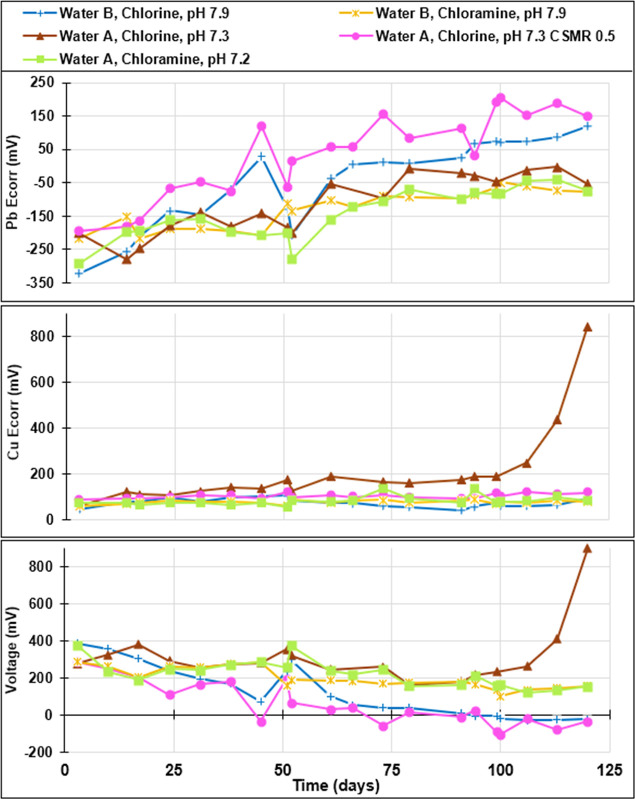
Phase 2 electrochemical corrosion potential (*E*_corr_) and voltage measured during flow at separated copper-solder couple. Slight anomalies in data trends near 50 days are associated with ∼3 days of elevated silica due to an exhausted ion exchange treatment column.

Given that only a few conditions occasionally demonstrated electrochemical reversal during flow in phase 1, a decision was then made to test lower pHs and CSMRs more similar to the previously cited Portland study for which we speculate that the electrochemical reversal of lead–tin solder might have occurred. During that second phase of experiments, a much more rapid rise in lead solder *E*_corr_ occurred in some cases without a corresponding rise in copper *E*_corr_, causing decisive and sustained electrochemical reversal. For instance, water B treated with chlorine caused solder *E*_corr_ to rise from −322 to +120 mV after only 120 days (Figure 3). This resulted in sustained electrochemical reversal from 94 days onward. The initial galvanic voltage of +386 mV decreased to −25 mV by day 120. As in phase 1, water A treated with free chlorine resulted in a dramatic rise of copper *E*_corr_, presumably due to copper pitting, and electrochemical reversal did not occur under continuous flow conditions. However, when additional sulfate was added to chlorinated water A at pH 7.3 to decrease the CSMR to a target of 0.5, the solder *E*_corr_ rose from −193 mV to as high as +205 mV. This caused sustained electrochemical reversal after 99 days with a galvanic voltage as low as −107 mV (Figure 3). Water A and water B never caused electrochemical reversal with chloramine (voltage was always >100 mV), nor did water A treated with orthophosphate (Supporting Information, Figure S1).

Galvanic current was also monitored during phase 1 (Supporting Information, Table S4) and phase 2 (Supporting Information, Table S5). Negative galvanic current protecting the lead solder from corrosion was recorded whenever a negative voltage occurred, confirming the nature of electrochemical reversal after prolonged exposure to chlorine.

### Lead Release and Electrochemistry of Simulated Lead Solder Joints During Stagnation

**Figure 4:**
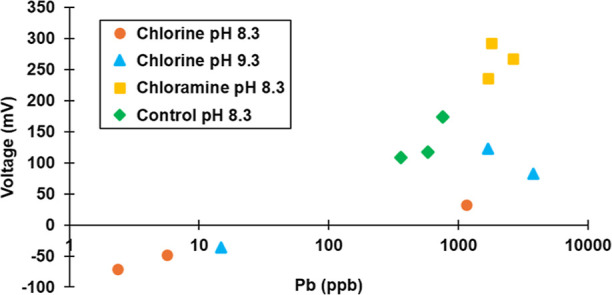
Phase 1 lead release vs voltage measured during stagnation for individual simulated joint replicates using water A.

During the water stagnation test following the phase 1 recirculating flow experiment, lead release and voltage (difference in *E*_corr_ of copper and solder surfaces) for each simulated joint replicate were measured (Figure 4). During stagnation, electrochemical reversal was documented for two out of three joints that were exposed to free chlorine at pH 8.3, and for one out of three joints exposed to free chlorine at pH 9.3. Electrochemical reversal never occurred for joints exposed to chloramine or those without a disinfectant.

In every case of electrochemical reversal for chlorine-treated joints, the lead release measured during stagnation was ≤15 ppb, which was over 2 orders of magnitude lower than for the comparable chloramine-treated joints (≥1700 ppb). Thus, even though free chlorine-induced electrochemical reversal was detected only once using the separated copper-solder wire apparatus during flow for these conditions, electrochemical reversal nonetheless occurred for three of the simulated joints during subsequent stagnation. Different behavior between flow and stagnation, or for a separated solder anode and copper cathode compared to a joint, is to be expected due to changes in corrosive microenvironments at the solder surface.^19,28^

Focusing on the nine results at target pH 8.3 (three joints treated with chlorine, three joints with chloramine, and three joints with no disinfectant), there was a reasonably strong correlation (*R*^2^ = 0.71) between lead release and galvanic voltage for individual joint replicates (Figure 4). There was a stronger linear relationship (*R*^2^ = 0.96) between the measured voltage of each joint and lead release in the presence of free chlorine (Figure 4). Similar to those at target pH 8.3, the one joint with a negative voltage at pH 9.3 had >100 times lower lead release than the other two replicates that had not yet demonstrated electrochemical reversal. We speculate that with months of additional exposure to high chlorine with frequent flow, electrochemical reversal would have eventually occurred for all replicates in the presence of free chlorine.

**Figure 5:**
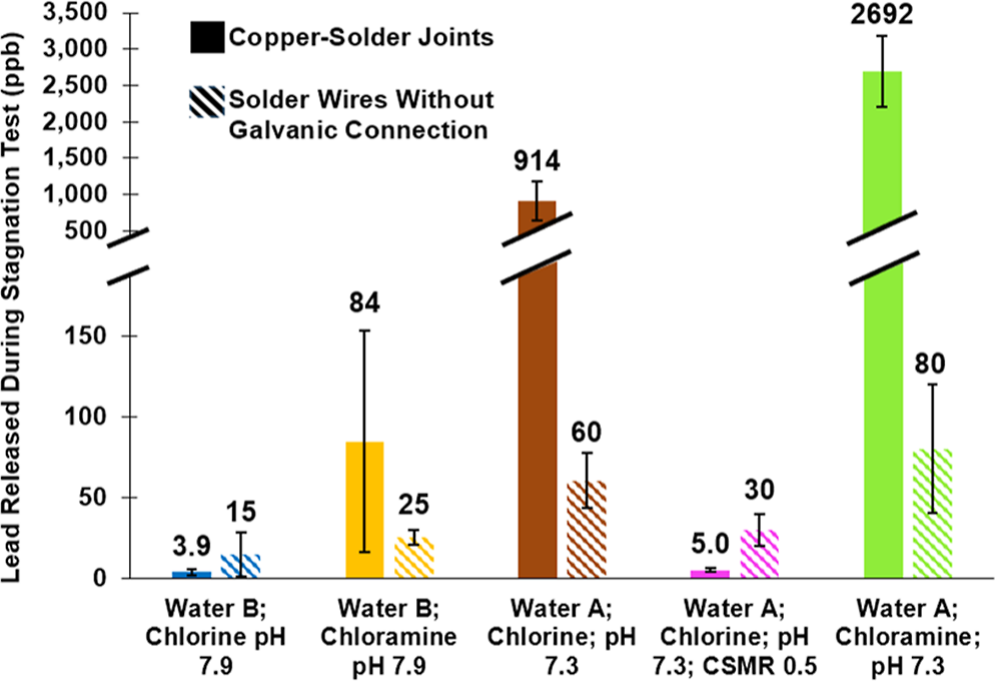
Lead release from phase 2 simulated joints during stagnation (solid bars) and solder wires without galvanic connection to copper (dashed bars). Error bars represent 95% confidence intervals from pooled data for triplicate joints sampled twice (*n* = 6) and from individual wires sampled six times (*n* = 6). Sustained electrochemical reversal was documented during flow for water B treated with chlorine (blue) and water A treated with chlorine and sulfate (pink).

A similar stagnation test for each individual joint was conducted after phase 2 (Figure 5). Water was collected daily, and a 3-day composite was analyzed for each joint. This sampling was conducted twice for all three replicate joints, and these data (*n* = 6) were pooled for statistical analysis. Unlike phase 1, when only some joints for each chlorinated condition demonstrated electrochemical reversal and low lead release (Figure 4), by the end of phase 2, lead release was low from every joint exposed to water conditions which demonstrated electrochemical reversal (Figure 5). For water B, lead release for joints exposed to free chlorine (3.9 ppb) was significantly lower (*p* = 0.013) than for joints exposed to chloramine (84 ppb), confirming the hypothesis that electrochemical reversal would dramatically decrease lead levels. Lead release was still significantly lower (*p* < 0.01) after exposure to chlorine (914 ppb) than to chloramine (2692 ppb) for joints treated with water A at pH 7.3, although electrochemical reversal was not measured in flow and lead levels remained relatively high compared to other treatment groups. When sulfate was added to chlorinated water A to decrease the CSMR to 0.5, lead levels plummeted to 5.0 ppb on average. Thus, lead release for this condition was about as low as water B treated with free chlorine, which also had electrochemical reversal during flow.

To determine whether the significant reductions in lead release in the presence of chlorine might also be consistent with the formation of a protective PbO_2_ scale, lead release from solder wires previously exposed to continuous flow in the separated galvanic couples was quantified at the same ratio of solder surface area to water volume. In each case where solder was anodic to copper, the solder wires without a connection to copper released on average 3–34 times less lead than the copper-solder joints, as expected if galvanic corrosion sacrificed the lead (Figure 5). Conversely, in each case where the solder wire had become cathodic to copper (e.g., electrochemical reversal), lead release was higher for the solder wires than that for the solder connected to copper, consistent with galvanic protection of the lead via electrochemical reversal.

Specifically, in the case of water B treated with chloramine, the galvanic connection to copper (e.g., simulated joints) caused average lead release >3 times the solder wire alone (*p* = 0.05). But for water B treated with free chlorine, average lead release was >3 times lower when the solder was galvanically connected to copper compared to the solder wire alone (*p* = 0.08). While the solder wire treated with chlorine did have 40% lower average lead release vs chloramine (15 ppb vs 25 ppb; *p* = 0.08), consistent with some hypothesized protective effect from Pb(IV) scale, this difference was trivial compared to the 22 times difference observed for the corresponding case with the galvanic connection to copper (3.9 ppb vs 84 ppb; *p* = 0.01). Thus, at least in this case, the galvanic effect was the dominant control on lead release (i.e., lead sacrificed or protected by connection to copper).

The presence of orthophosphate with chlorine resulted in significantly higher (*p* < 0.01) lead release for water A versus chlorine alone from simulated joints (Supporting Information, Figure S2). This was consistent with expectations that orthophosphate may sometimes interfere with the corrosion inhibiting effects of chlorine for lead release.^8,27^

Tin levels from the joint stagnation test were generally much lower than lead levels (Supporting Information, Figure S3), and joints that experienced electrochemical reversal also tended to release slightly less tin, although the results were not as significant as those for lead. In addition, when electrochemical reversal occurred in the case of water B, copper release was slightly (18%) higher when it was sacrificed, compared to conditions where it was protected from corrosion by anodic lead, although the effect was not statistically significant (Supporting Information, Figure S4; *p* = 0.34). This small effect for copper is consistent with the low magnitude of the galvanic current after reversal occurred (Supporting Information, Table S5) and with the greater surface area for copper than lead in a joint (i.e., there is >5 times more copper than lead–tin on the surface of the simulated joint).^29^ On the other hand, water A treated with chlorine and additional sulfate to increase the CSMR to 0.5 resulted in >5 times higher copper release (*p* < 0.01) than water A treated with chloramine and without additional sulfate. Aside from the difference in chlorine vs chloramine, this may be in part due to higher sulfate levels, which have been shown to increase copper corrosion rates.^30^

### Solder Surface Analysis

**Figure 6:**
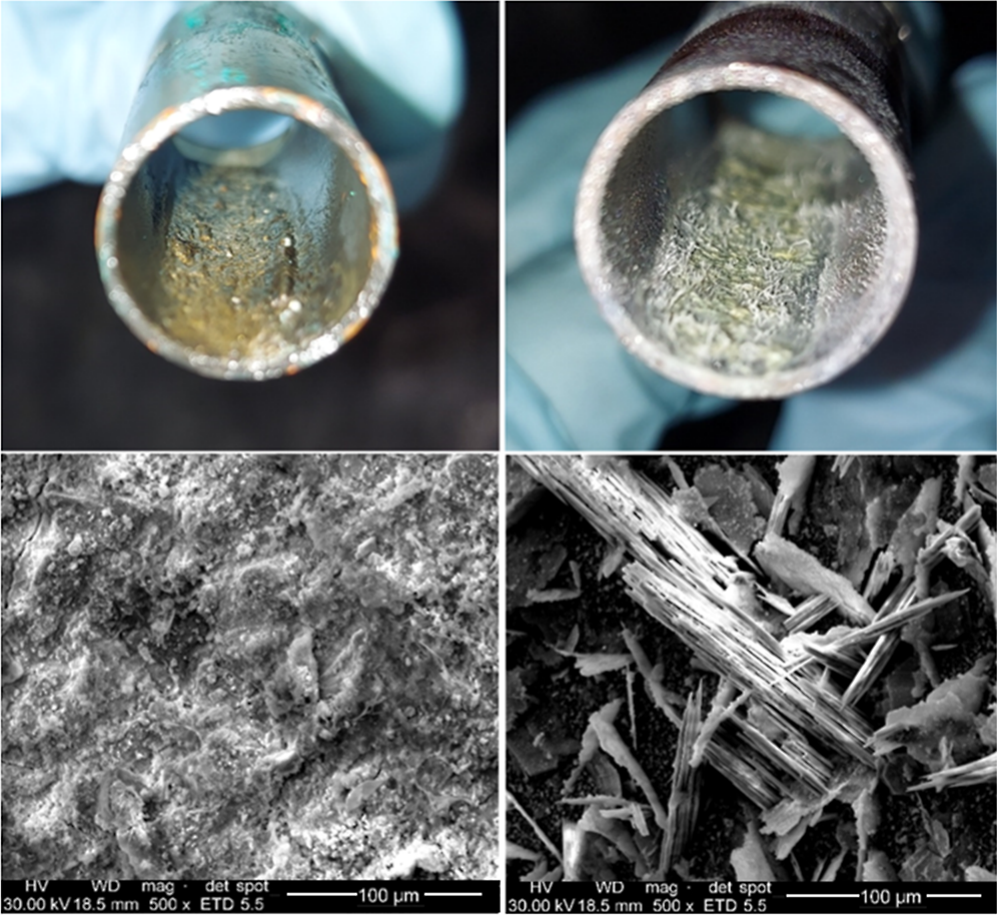
Lead–tin solder surface of select joints treated with chlorine (left) and chloramine (right), taken with a camera (top) and with SEM at 500× magnification (bottom).

The solder surfaces of two joints treated with water A at pH 8.3 and exposed to either free chlorine or chloramine were photographed using both a camera and SEM at 500× magnification (Figure 6). Based on the aforementioned stagnation test, the selected chlorine-treated joint had demonstrated electrochemical reversal, with a negative voltage (−48 mV) and low lead release during stagnation (5.6 ppb). Voltage and lead levels remained high for the corresponding chloramine-treated joint (+235 mV and 1700 ppb, respectively). Both to the naked eye and using SEM at 500× magnification, the solder surface appeared considerably smoother for the joint treated with free chlorine, consistent with passivation and its dramatically lower lead release. While the percentage of lead on the scale of the solder surfaces treated with chlorine vs chloramine were similar based on SEM measurements (Supporting Information, Table S6), their appearance and mineralogical composition differed. The solder surface of the chlorine-treated joint was brownish–black in color, consistent with the formation of Pb(IV), which was further supported by XRD analysis of the solder surface (Supporting Information, Figure S5). In contrast to pure lead pipe, a lead–tin alloy connected to copper constitutes a more complicated system. Focusing on lead, peaks corresponding to plattnerite (PbO_2_-β) were detected on the solder surface of chlorine-treated joints. Prior work with pure lead pipes also showed that plattnerite would form more readily than scrutinyite (PbO_2_-α) after exposure to chlorine.^10^ Such peaks were not detected on chloramine-treated joints (Supporting Information, Figure S5), consistent with visual observations, elevated lead release, and the expectation that Pb(IV) would form only after prolonged exposure to free chlorine. Various other peaks were detected, and some were not able to be identified, which is expected due to the system’s complexity.

### Implications

These novel results may help explain a range of water lead contamination issues observed, or not observed, at certain water utilities. Electrochemical reversal provides a viable explanation for extremely low lead release reported previously for free chlorine versus chloramine in Portland and the neighboring city of Tigard, OR. Although their 1980s pilot study demonstrated that free chlorine caused >10 times less lead release than chloramine after about 8 months, Portland nonetheless used chloramine without a phosphate-based corrosion inhibitor for several decades and exceeded the 15 ppb lead action level 11 times since 1997.^31^ Portland has no lead service lines, and its ongoing problems with elevated water lead levels due to solder corrosion have generated controversy and media attention over the past 20 years.^32−36^ Likewise, Tigard, OR, exceeded the 15 ppb lead action level at least eight times from 1997 to 2016 while using chloraminated water purchased from Portland.^37,38^ But after switching to a different low alkalinity source water treated with free chlorine,^37,39^ Tigard’s 90th percentile lead levels plummeted from 13.0 to 0.0 ppb within a year,^38^ a result that is consistent with Portland’s pilot study results from the 1980s and data presented herein.^21^ Another well-controlled laboratory study using a low alkalinity and circumneutral pH water also demonstrated an order of magnitude lower lead release for chlorine vs chloramine from copper pipe rigs with lead–tin solder.^40^ These laboratory and field observations are consistent with hypothesized formation of PbO_2_ due to free chlorine.^41^

Conversely, a possible loss of electrochemical reversal for solder after switching from free chlorine to chloramine might also explain a mysterious spikes of lead observed in Brick, NJ in 2014,^16^ and partly explain similar problems observed in Greenville, NC in 2006.^15^ At both utilities, lead release was very low using free chlorine and rose dramatically after switching to chloramine. Another recent survey also indicated that many utilities with water normally considered corrosive to solder due to high CSMR did not have noteworthy lead problems: several of these utilities were determined to be using free chlorine.^42^

The fact that free chlorine might be inhibiting lead solder contamination of water without a utility’s knowledge is an important discovery that should be considered during desktop and experimental studies of corrosion control, especially when changes in disinfectant or source waters are considered.^11,43−45^ Traditional jar studies of lead solder corrosion, while very useful in reproducing many trends observed for solder corrosivity, are unlikely to reveal electrochemical reversal since it can require a few months or longer to occur even during the continuous flow and high chlorine conditions tested herein. Electrochemical reversal might require years or decades to occur at lower levels of chlorine during jar testing without flow. It is also likely that CSMR, organic matter, alkalinity, pH, and other factors would affect electrochemical reversal of solder joints since these factors are known to affect formation of Pb(IV) on lead pipe and the galvanic corrosion of Pb/Sn solder.^46−49^ Further, the alkalinity tested in these experiments was low (<20 mg/L as CaCO_3_) and the previously cited case studies for which we suspect electrochemical reversal to have occurred also involved relatively low alkalinity waters. Future work would be beneficial to evaluate whether high alkalinity may accelerate or decelerate this phenomenon.

We also note that lead contamination from solder at its most extreme can cause contamination worse than that from prior high-profile events associated with lead pipes. For example, 90th percentile lead values recorded at a recent incident of solder corrosion were higher than those reported in Flint, MI or even Washington, DC.^18^ Additionally, a new building studied by Lytle et al. in the 1990s demonstrated sporadic lead release over 1000 ppb for numerous faucets associated with Pb/Sn solder.^50^ When it is further considered that roughly 80 million residences likely have lead solder, versus a few million homes with lead service line pipe,^51^ our present lack of understanding of lead contamination from solder corrosion is worrisome. The discovery of electrochemical reversal of lead–tin solder and copper in this work, and the recent finding that nitrate can trigger severe galvanic corrosion,^18^ have demonstrated that present knowledge regarding lead solder corrosion is inadequate to properly protect public health.

## Supporting Information Available

Additional experimental details, XRD analysis, galvanic current data, tin and copper release, and impact of orthophosphate (PDF)
