# Supplementary material for: Free Chlorine Can Inhibit Lead Solder Corrosion via Electrochemical Reversal
Source: Environ Sci Technol. 2024 Oct 18;58(43):19454–61. doi: 10.1021/acs.est.4c07375 (PMC11526352; doi:10.1021/acs.est.4c07375)
Supplement: Supplementary file 1 — es4c07375_si_001.pdf [file es4c07375_si_001.pdf]

Free Chlorine Can Inhibit Lead Solder Corrosion via Electrochemical Reversal

Frank A. Mazzola[1*#], Kathryn G. Lopez[1†*], and Marc Edwards[1]

[1]The Charles Edward Via, Jr. Department of Civil and Environmental Engineering, Virginia Tech, Blacksburg, Virginia 24061, United States
[†]Present Address for K.L.: AAAS Science and Technology Policy Fellowship Programs Inc., U.S. Congress, Washington, DC 20002
[*]Denotes co-first authors
[#]Corresponding author: frank16@vt.edu

Summary: 7 pages, 6 tables, 5 figures.

Supporting Information

SI Table 1. General Water Chemistries 2

SI Table 2. Lead release from soldered joints during conditioning in Water A 2

SI Table 3. Supplemental water conditions and target parameters 2

SI Table 4. Galvanic current measured at separated copper-solder couple during Phase 1........3

SI Table 5. Galvanic current measured at separated copper-solder couple during Phase 2........4

SI Table 6. Atomic percentage of lead, tin, and copper on the solder surface of select chlorine and chloramine-treated joints from phase 1. Plus-minus values represent one standard deviation based on 10 measurements from different locations on the solder surface using SEM. 4

SI Figure 1. Phase 1 electrochemical corrosion potential (E_{corr}) and voltage from separated copper-solder couple for supplemental conditions 5

SI Figure 2. Lead release for free chlorine-treated joints with and without orthophosphate.........6

SI Figure 3. Tin release from soldered joints during Phase 2 stagnation test 6

SI Figure 4. Copper release from soldered joints during Phase 2 stagnation test 7

SI Figure 5. XRD analysis of solder surface from selected Phase 1 joints treated with free chlorine or chloramine (Water A at pH 8.3). Reference XRD patterns were obtained from the International Centre for Diffraction Data. 7

SI Table 1. General Water Chemistries

	Calcium (mg/L)	Magnesium (mg/L)	Silica (mg/L as Si)	Chloride (mg/L)	Sulfate (mg/L)	Alkalinity (mg/L as $CaCO_3$)
Water A	1.4	0.2	3.7	10	1.0	12
Water B	1.8	1.2*	5.9	10	5.0*	17

**Condition with adjusted CSMR of 0.5 had ~5 mg/L Mg^{2+} and ~20 mg/L SO_4^{2-}*

SI Table 2. Lead release from soldered joints during conditioning in Water A

Phase	Test Water Name	Mean Pb Release (ppb)	Standard Deviation of Pb Release (ppb)
1	Water A, Chlorine, pH 8.3	1240	254
	Water A, Chlorine, pH 9.3	1280	174
	Water A, Chlorine, pH 9.3, CSMR 0.5	1280	259
	Water A, Chlorine, pH 9.3, CSMR 0.5, 1 mg/L PO_4^{3-}	1240	244
	Water A, Chloramine, pH 8.3	1290	185
	Water A, Control, pH 8.3	1240	232
2	Water B, Chlorine, pH 7.9	1090	159
	Water A, Chlorine, pH 7.3	1100	112
	Water A, Chlorine, pH 7.3, CSMR 0.5	1110	224
	Water A, Chlorine, pH 7.3, 1 mg/L PO_4^{3-}	1110	119
	Water B, Chloramine, pH 7.9	1090	216
	Water A, Chloramine, pH 7.2	1090	174

SI Table 3. Supplemental water conditions and target parameters

Test Water Name	Water	pH	Chlorine (mg/L as Cl_2)	Orthophosphate (mg/L as PO_4^{3-})	Approx. CSMR
Water A, Chlorine, pH 9.3, CSMR 0.5	A	9.3	4.0	0	0.5
Water A, Chlorine, pH 9.3, CSMR 0.5, 1 mg/L PO_4^{3-}	A	9.3	4.0	1.0	0.5
Water A, Chlorine, pH 7.3, 1 mg/L PO_4^{3-}	A	7.3	4.0	1.0	10

SI Table 4. Galvanic current measured at separated copper-solder couple during Phase 1

Time (days)	Water A, Chlorine, pH 8.3	Water A, Chlorine, pH 9.3	Water A, Chloramine, pH 8.3	Water A, Control, pH 8.3
	Galvanic Current (µA)			
3	4.79	23.7	2.14	0.54
10	5.03	15.0	5.82	0.85
17	5.22	14.2	3.36	2.51
24	2.22	1.32	3.68	1.29
31	3.78	9.19	5.77	2.10
37	2.84	11.7	3.46	1.28
45	1.77	19.0	3.12	2.04
52	1.85	8.59	2.68	1.44
59	1.84	10.9	2.83	2.66
66	1.96	11.3	2.57	3.71
73	4.72	9.74	2.25	3.80
80	8.23	7.02	1.87	7.22
87	6.63	5.92	2.05	11.9
94	3.11	7.90	1.34	12.1
101	3.61	5.51	1.23	6.11
108	3.01	8.48	1.48	4.33
115	13.4	3.27	1.60	5.49
122	39.2	2.5	1.38	9.40
129	28.7	5.59	0.93	8.18
136	27.3	9.22	0.90	4.35
143	21.2	2.33	0.99	3.89
150	29.3	2.98	0.33	1.54
157	51.5	2.71	1.33	8.23
164	23.3	1.37	0.89	1.92
171	26.1	1.11	0.91	1.35
178	15.6	0.85	0.56	1.08
185	18.2	1.77	0.86	2.88
192	40.3	4.62	0.79	1.52
199	37.3	2.12	1.43	1.29
206	38.7	2.27	0.82	3.45
213	34.9	1.24	1.11	6.09
220	43.4	1.44	1.89	2.98
227	36.8	-0.32	2.12	3.98
241	52.4	1.76	1.21	1.67
248	46.3	2.61	1.65	0.87
255	29.0	1.70	1.16	1.22

SI Table 5. Galvanic current measured at separated copper-solder couple during Phase 2

Time (days)	Water B, Chlorine, pH 7.9	Water B, Chloramine, pH 7.9	Water A, Chlorine, pH 7.3	Water A, Chlorine, pH 7.3, CSMR 0.5	Water A, Chloramine, pH 7.2
	Galvanic Current (μA)				
3	7.6	1.5	5.4	6.5	4.2
10	5.5	1.2	4.6	4.2	1.3
17	2.1	1.0	6.6	3.3	3.7
24	1.4	1.5	7.5	0.7	2.2
31	0.82	1.2	4.0	2.3	1.8
38	1.5	2.2	3.4	4.3	6.2
45	0.28	2.3	6.6	-0.03	2.1
51	0.75	0.10	15	2.3	1.5
52	3.5	0.12	5.6	0.13	1.4
61	0.86	0.10	3.0	-0.05	1.3
66	0.34	0.34	not recorded	0.27	0.92
73	0.3	0.23	4.6	-0.09	0.81
79	0.2	0.37	1.4	-0.13	0.68
91	0.05	0.17	1.7	-0.10	0.43
99	0.04	0.19	3.8	-0.26	0.41
100	-0.04	0.09	not recorded	-0.26	0.44
106	-0.04	0.16	3.3	-0.08	0.35
113	-0.05	0.10	9.0	-0.20	0.22
120	-0.05	0.15	20	-0.05	0.28

SI Table 6. Atomic percentage of lead, tin, and copper on the solder surface of select chlorine and chloramine-treated joints from phase 1. Plus-minus values represent one standard deviation based on 10 measurements from different locations on the solder surface using SEM.

	Pb	Sn	Cu
**Chlorine**	9.7% ± 3.9%	6.0% ± 1.7%	6.2% ± 3.3%
**Chloramine**	7.9% ± 5.4%	7.6% ± 3.2%	8.3% ± 13%

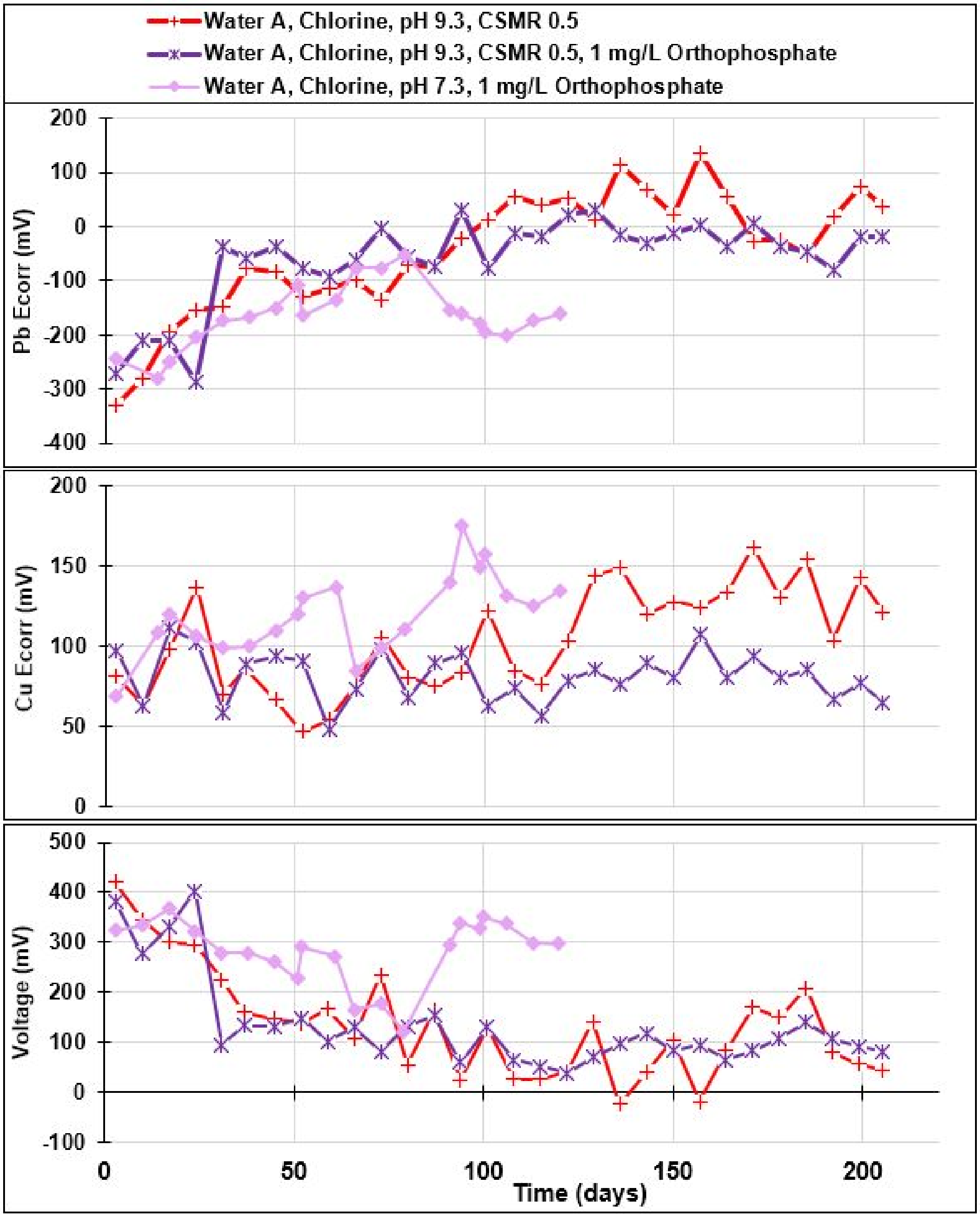

SI Figure 1. Phase 1 electrochemical corrosion potential (E_{corr}) and voltage from separated copper-solder couple for supplemental conditions

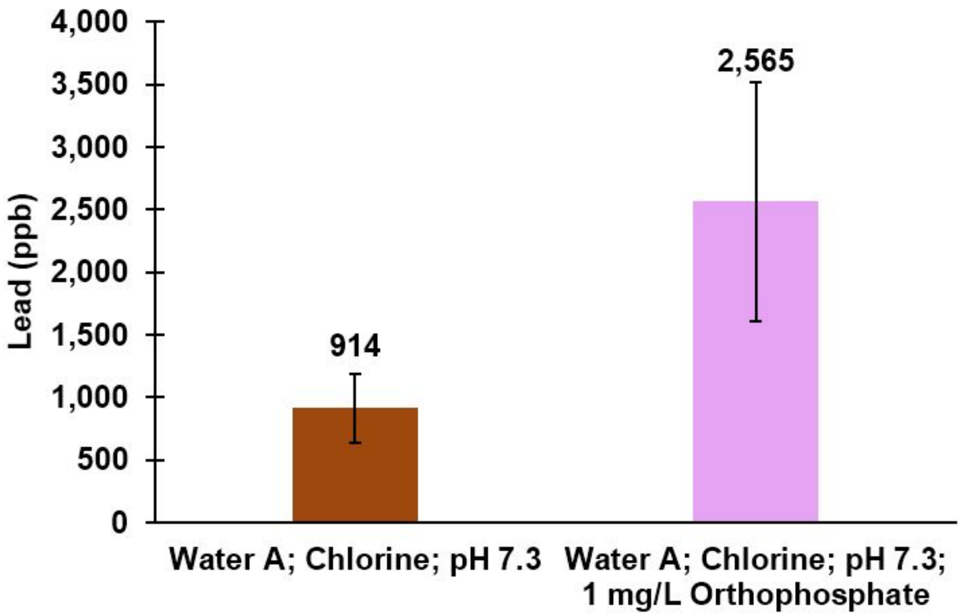

SI Figure 2. Lead release for free chlorine-treated joints with and without orthophosphate

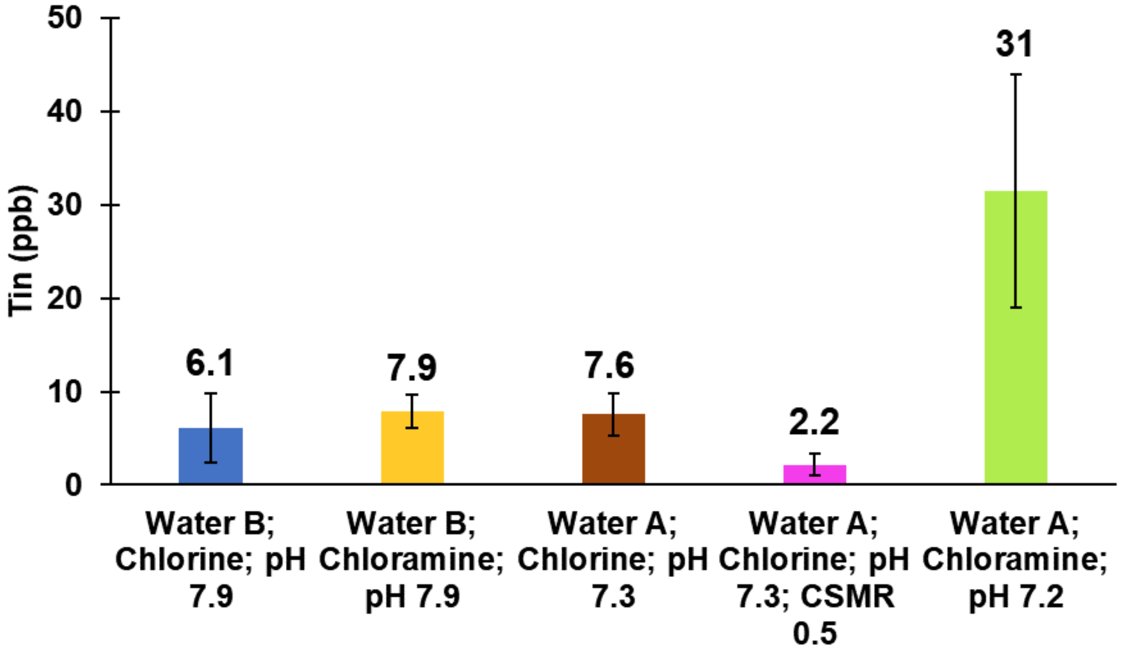

SI Figure 3. Tin release from soldered joints during Phase 2 stagnation test

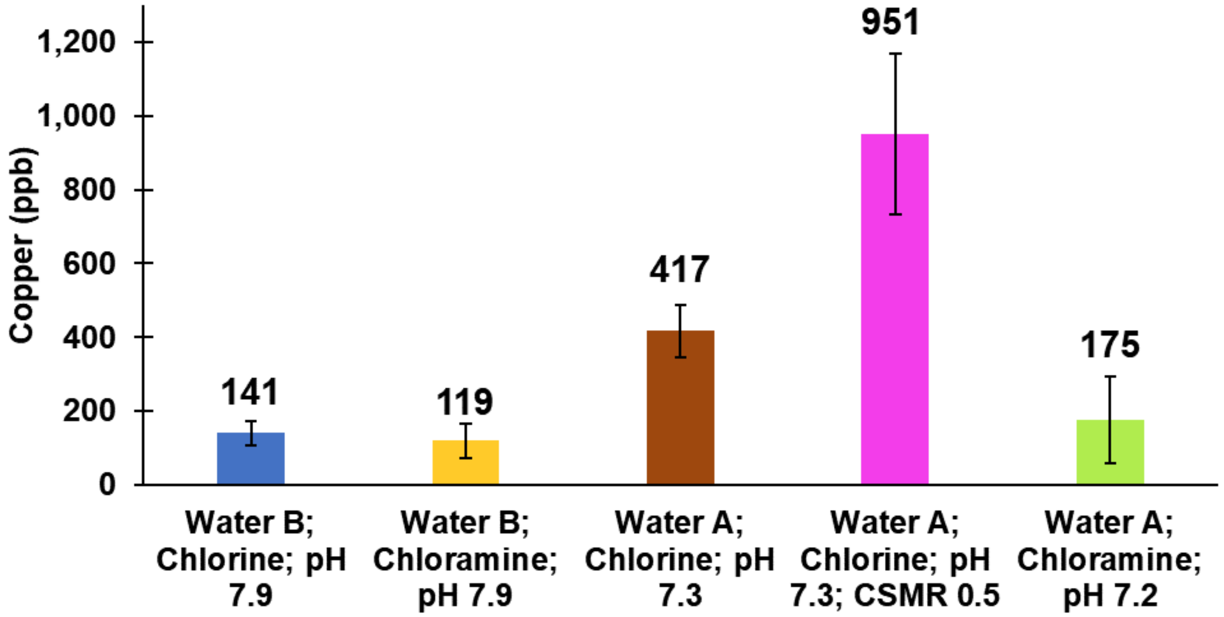

SI Figure 4. Copper release from soldered joints during Phase 2 stagnation test

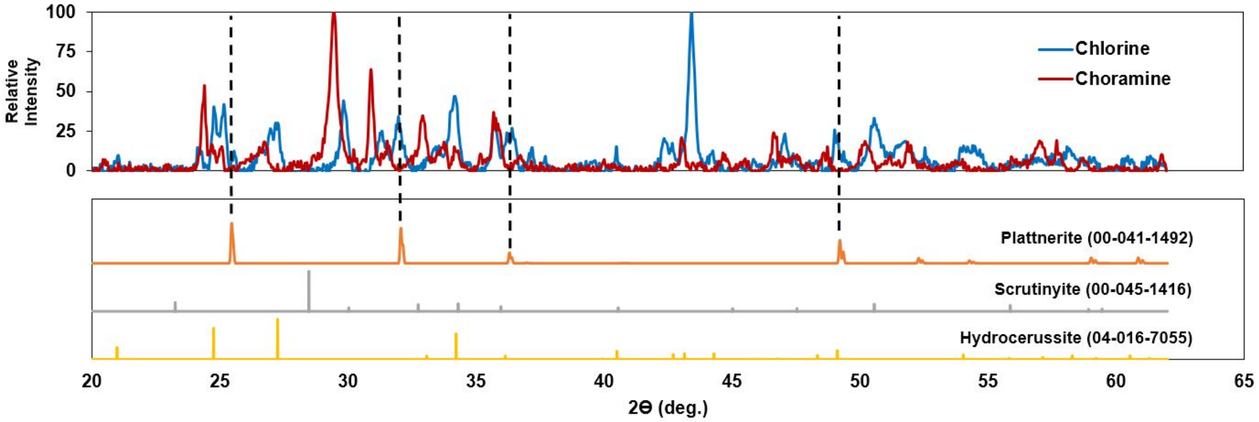

SI Figure 5. XRD analysis of solder surface from selected Phase 1 joints treated with free chlorine or chloramine (Water A at pH 8.3). Reference XRD patterns were obtained from the International Centre for Diffraction Data.
